# An Evaluation of the Effects of the Novel Antipsychotic Drug Lurasidone on Glucose Tolerance and Insulin Resistance: A Comparison with Olanzapine

**DOI:** 10.1371/journal.pone.0107116

**Published:** 2014-09-25

**Authors:** Claire Wu, Jessica Yuen, Heidi N. Boyda, Ric M. Procyshyn, Cathy K. Wang, Yahya I. Asiri, Catherine C. Y. Pang, William G. Honer, Alasdair M. Barr

**Affiliations:** 1 Department of Pharmacology, University of British Columbia, Vancouver, British Columbia, Canada; 2 Department of Psychiatry, University of British Columbia, Vancouver, British Columbia, Canada; 3 British Columbia Mental Health and Addictions Research Institute, Vancouver, British Columbia, Canada; University of Texas Health Science Center at San Antonio, United States of America

## Abstract

Over the past two decades, there has been a notable rise in the use of antipsychotic drugs, as they are used to treat an increasing number of neuropsychiatric disorders. This rise has been led predominantly by greater use of the second generation antipsychotic (SGA) drugs, which have a low incidence of neurological side-effects. However, many SGAs cause metabolic dysregulation, including glucose intolerance and insulin resistance, thus increasing the risk of cardiometabolic disorders. The metabolic effects of the novel SGA lurasidone, which was approved by the Food and Drug Administration in 2010, remain largely unknown. As rodent models accurately predict the metabolic effects of SGAs in humans, the aim of the present study was to use sophisticated animal models of glucose tolerance and insulin resistance to measure the metabolic effects of lurasidone. In parallel, we compared the SGA olanzapine, which has established metabolic effects. Adult female rats were treated with vehicle, lurasidone (0.2, 0.8 or 2.0 mg/kg, s.c.) or olanzapine (10.0 mg/kg, s.c.) and subjected to the glucose tolerance test (GTT). Separate groups of rats were treated with vehicle, lurasidone (0.2, 0.8 or 2.0 mg/kg, s.c.) or olanzapine (1.5 and 15 mg/kg, s.c.) and tested for insulin resistance with the hyperinsulinemic-euglycemic clamp (HIEC). Compared to vehicle treated animals, lurasidone caused mild glucose intolerance in the GTT with a single dose, but there was no effect on insulin resistance in the GTT, measured by HOMA-IR. The HIEC also confirmed no effect of lurasidone on insulin resistance. In contrast, olanzapine demonstrated dose-dependent and potent glucose intolerance, and insulin resistance in both tests. Thus, in preclinical models, lurasidone demonstrates mild metabolic liability compared to existing SGAs such as olanzapine. However, confirmation of these effects in humans with equivalent tests should be confirmed.

## Introduction

Second generation antipsychotic (SGA) drugs are widely used for the treatment and management of schizophrenia and other psychiatric disorders [1]–[3]. The use of SGAs has increased substantially in recent years, and the 1-year prevalence of antipsychotic drug use now exceeds 3% of the population in some countries [4]. While SGAs have a lower likelihood of neurological side-effects than the first generation antipsychotic drugs, they are frequently associated with serious cardiometabolic side-effects [5]–[8]. This has led to the development of newer SGA drugs with the goal of improving tolerability and reducing the risk of adverse events, such as weight gain, insulin resistance, glucose intolerance and dyslipidemia. These sequelae are established components of metabolic syndrome, which predisposes patients to secondary diseases, such as Type 2 Diabetes Mellitus (DM) and cardiovascular disease [9]. The incidence of SGA-induced metabolic side-effects is high [10]. For example, in a large head-to-head clinical trial of SGA drugs, the Clinical Antipsychotic Trial of Intervention Effectiveness (CATIE) study [a major, multi-center trial sponsored by NIMH] observed that 43% of patients treated with SGAs had metabolic syndrome. When controlling for BMI, CATIE men were 85%, and CATIE women 137% more likely to have metabolic syndrome than non-psychiatric counterparts [11].

Within the SGA class, there is a wide spectrum with regards to the risk of metabolic side-effects, which is most commonly measured by weight gain. Head-to-head comparisons have confirmed that the drugs olanzapine and clozapine cause greatest weight gain [12]–[14], followed by drugs such as risperidone and quetiapine, while more “weight-neutral” drugs including ziprasidone and aripiprazole have notably fewer metabolic side-effects. However, the risk for hyperglycemia and insulin resistance associated with SGAs may be independent of effects on body weight [15]. Numerous reports have detailed hyperglycemia and new-onset diabetes in the absence of substantial weight gain in SGA-treated patients [14]. Furthermore, studies in non-psychiatric subjects have shown that the same SGAs that cause metabolic dysregulation in patients also cause rapid-onset glucose intolerance before major weight gain can occur [16], [17]. For example, in a recent double-blind, placebo-controlled crossover trial of olanzapine in non-psychiatric subjects, 3-day treatment with a clinical dose of olanzapine caused significant impairments in the glucose tolerance test [18]. Another recent study with non-psychiatric subjects used the hyperinsulinemic euglycemic clamp (HIEC) to show that treatment with olanzapine for nine days caused significant insulin resistance in the absence of weight gain, increases in food intake, or hunger [19].

Rodent models reliably parallel many aspects of the metabolic dysregulation caused by SGA treatment in humans [20]. Most animal models show findings consistent with clinical studies, as SGA drugs that cause greater metabolic dysregulation in humans result in more pronounced metabolic effects in rodent paradigms [21]–[33]. With a strong predictive validity, these models are therefore useful in evaluating the metabolic side-effects of novel antipsychotic drugs where a more thorough metabolic evaluation has not been conducted in humans, due to the invasive nature of the required procedures. In October 2010, the novel SGA lurasidone was approved by the US Food and Drug Administration for the treatment of adults with schizophrenia, and subsequently for bipolar disorder (June 2013) [34]. Studies have generally reported that lurasidone is well tolerated in clinical trials, with a lower incidence of weight gain than most current SGAs [35], [36]. However, to our knowledge, there has not yet been an evaluation of lurasidone with regards to the potential side-effects of glucose intolerance and insulin resistance, in any species. The goal of the current study was therefore to evaluate the effects of lurasidone on these two key indices of metabolic health, across a wide range of doses, using “gold-standard” techniques such as the HIEC. As a comparator, we concurrently measured the metabolic effects of olanzapine, which is a drug with established metabolic liability [36].

## Methods and Materials

### Animals

Adult, female Sprague-Dawley rats weighing 250–275 g (Charles River, Montreal, Canada) were maintained under a 12-hour light/dark cycle (lights on at 07∶00 h) at 22±1°C and habituated to the UBC colony one week prior to experimentation. Rats were pair-housed and allowed free access to food and water. Female rats were used as they tend to show antipsychotic-drug induced metabolic disturbances that are more consistent and robust than males [20], [22]. Animals were treated in accordance with the NIH Guidelines for the Care and Use of Laboratory Animals; all procedures were approved by the UBC Animal Care and Use Committee.

### Pharmacological Agents and Solutions

Lurasidone and olanzapine [Toronto Research Chemicals Inc., Toronto, ON] were formulated in 50% polyethylene glycol 400, 40% distilled water and 10% ethanol (PEG solution), as previously [37]–[39]. All drug solutions were dissolved at a volume of 1 ml/kg on the day of the experiment. Recombinant human insulin (Humulin R) [Eli Lily, Indianapolis, IN] and dextrose (50%) were prepared using 0.9% saline.

### Intraperitoneal Glucose Tolerance Test (IGTT) (see Figure 1A for experimental protocol)

Rats were fasted overnight (16±2 hours) and randomly assigned to one of five treatment groups: lurasidone (0.2, 0.8, 2 mg/kg, s.c.), olanzapine (10 mg/kg, s.c.) and vehicle (PEG solution, s.c.) [n = 8 per group]. Blood glucose measurements were determined using a hand-held glucometer (One Touch Ultra) by wrapping the rat in a towel and producing a drop of blood from the saphenous vein with a 25-gauge needle. After measuring the baseline fasting blood glucose at time = 0 minutes, animals received one of the five treatments via a single s.c. injection. A second glucose measurement was taken at t = 30 minutes to assess the effect of drug treatment on the fasting blood glucose level. Subsequently, a saphenous blood draw using heparinized collecting tubes was performed to obtain plasma samples for analysis of insulin levels; extracted blood samples were centrifuged (10,000 RPM, 10 Min, 4°C) and samples stored at −80°C. This was followed by a glucose challenge (1 g/kg/ml, i.p.), marking the start of the IGTT. Blood glucose readings were then repeatedly determined every 15 minutes for the next 2 hours. Animal handlers were blinded to the treatment conditions.

**Figure 1:**
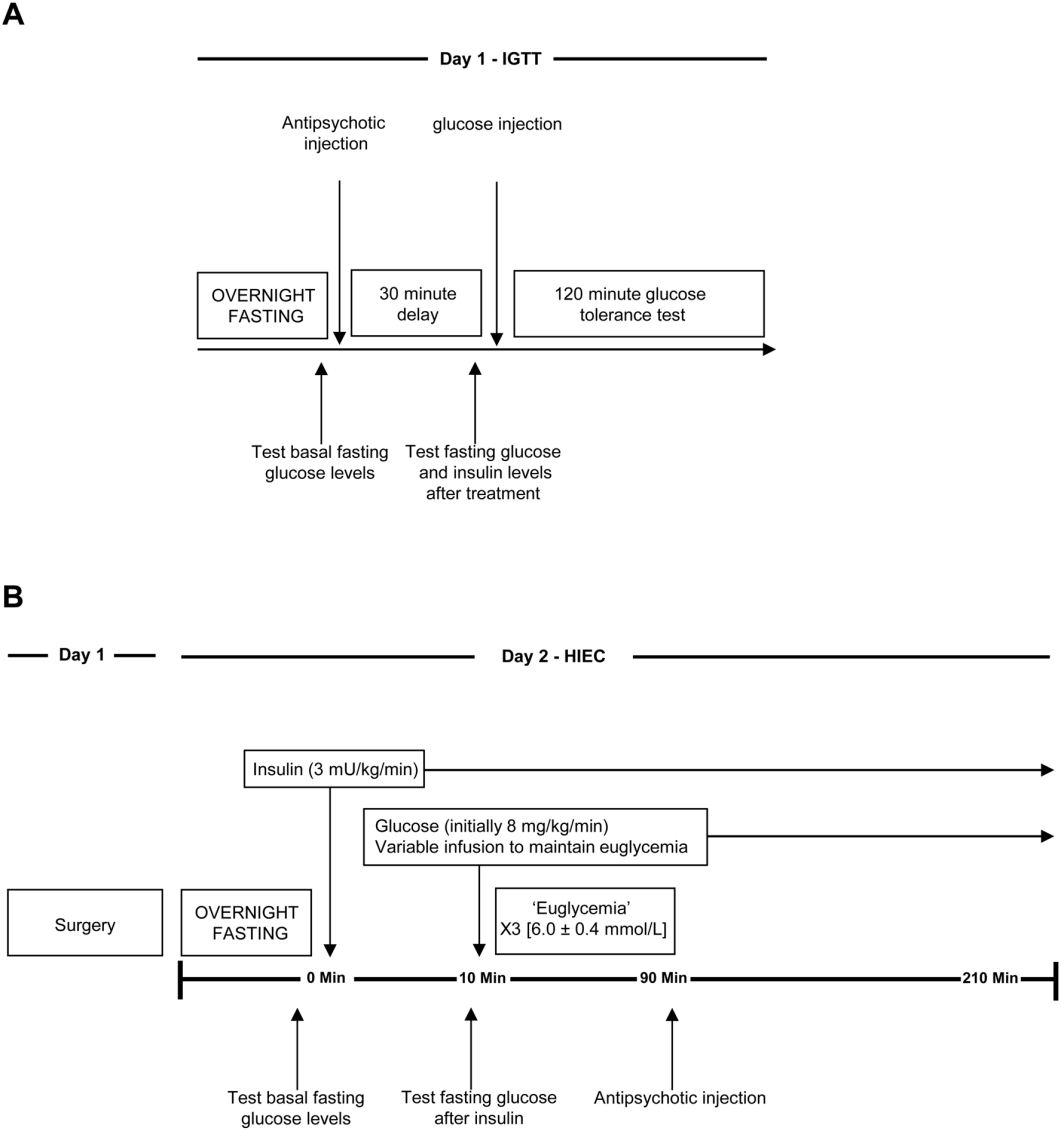
Experimental protocol. Describing (**A**) the intraperitoneal glucose tolerance test and (**B**) the hyperinsulinemic-euglycemic clamp with acute antipsychotic drug treatment.

### Surgical Preparations for Hyperinsulinemic-Euglycemic Clamp (HIEC)

Rats were anaesthetized with isoflurane and administered ketoprofen pre-operatively (5 mg/kg, s.c.), while bupivacaine was applied to the incision sites. The left common carotid artery and both exterior jugular veins were catheterized using polyethylene cannulae (PE50) filled with heparinized saline (25 IU/ml heparin). The arterial cannula was used to sample blood for determination of blood glucose, while the jugular veins were for constant infusion of insulin and variable infusion of dextrose. All cannulas were tunneled subcutaneously and exteriorized through an incision in the back of the neck. Rats were recovered overnight.

### HIEC Procedures (see Figure 1B for experimental protocol)

Rats were fasted overnight (16±2 hours) prior to the onset of the HIEC. Insulin (3 mU/kg/min) and dextrose (50% w/v) were infused through the venous cannulas using infusion-only pumps (Harvard Apparatus, Holliston, MA). Insulin infusion was started and continued at a constant rate throughout the experiment after taking a baseline fasting blood glucose reading from the arterial cannula using a hand-held glucometer (One Touch Ultra). After 10 minutes, dextrose was infused at an initial rate of 8 mg/kg/min (0.96 ml/kg/hr) and the glucose infusion rate (GIR) was adjusted accordingly to achieve euglycemia based on glucose readings taken every 10 minutes. Three consecutive blood glucose readings in the range of 6.0±0.4 mmol/L at a single GIR indicated euglycemia. At this point, animals randomly received one of the following treatments via a single s.c. injection: lurasidone (0.2, 0.8 and 2.0 mg/kg), olanzapine (1.5, 15 mg/kg) and vehicle (PEG solution) [n = 6–10 per group]. The GIR was adjusted appropriately to maintain euglycemia for the next two hours. Animal handlers were blinded to drug treatments.

### Plasma Insulin Measurement by ELISA

Insulin levels were measured using the ultra-sensitive rat insulin ELISA kit (Crystal Chem Inc., IL, USA). Plasma samples (5 µl) were added and analyzed, in duplicate, on 96 well plates. Samples were incubated, followed by repeated washes. Substrate was added and absorbance measured at 450 nm–630 nm, as previously [40], [41]. Calibrators were prepared and used to generate a calibration curve. A reference (non-fasted) animal’s plasma added to all plates served as reference standard; confirming a high intra-plate reliability. One animal’s plasma sample was spoiled (olanzapine treated) and not used for analysis.

### Insulin resistance

Determination of insulin resistance in rats was accomplished using the homeostatic model assessment of insulin resistance (HOMA-IR) equation (1) [42]. The product of both the fasting levels of glucose (expressed as mmol/L) and insulin (µU/ml) 30 minutes post-drug administration is divided by a constant of 22.5. Greater insulin resistance is represented via a larger calculated HOMA-IR score.
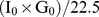
(1)where I_0_ and G_0_ are fasting insulinemia and glycaemia.

### Statistical analysis

Metabolic indices during the IGTT were analyzed by one-way analysis of variance (ANOVA), with drug dose as the between group factor. For the IGTT, glucose data were summed as the area-under-the-curve throughout the 120 minute procedure [37]. For the HIEC data, a between-within subject analysis was performed, with drug treatment as the between subjects factor and change in GIR from baseline as the within factor. Alpha value was set at *p*<0.05. LSD post-hoc tests were conducted when a main effect or interaction between main effects was significant. Data were analyzed with SPSS software, Chicago, IL, version 21.

## Results

### IGTT

Analysis of the IGTT with lurasidone and olanzapine revealed no difference between fasting glucose levels either before or after drug treatment compared to vehicle treated rats. There was, however, a significant main effect of drug treatment on fasting insulin levels [F_(4,39)_ = 6.02, p = 0.001], whereby olanzapine selectively increased insulin levels to more than twice those of any other group (p<0.001) (Table 1). A similar effect was measured with insulin resistance calculated by the HOMA-IR equation, whereby there was a significant main effect of drug treatment [F_(4,39)_ = 5.43, p<0.005] as the olanzapine treated group showed a significant increase in insulin resistance compared to all other groups (p<0.001) and no effect of lurasidone treatment (Table 1).

**Table 1:** Mean concentration of fasting glucose, insulin and HOMA-IR scores in antipsychotic drug treated rats.

Antipsychotic Drug	Measure	Treatment Dose (mg/kg)			
		0	0.2	0.8	2
**Lurasidone**	**G_0_**	5.6±0.1	5.4±0.3	5.3±0.2	5.2±0.2
	**I_0_**	10.0±0.7	10.4±0.8	9.5±0.7	10.4±0.9
	**HOMA-IR**	2.5±0.2	2.5±0.2	2.2±0.8	2.5±0.3
					**10**
**Olanzapine**	**G_0_**				5.5±0.2
	**I_0_**				22.9±5.4*
	**HOMA-IR**				5.8±1.5*

I_0_ = fasting insulin levels (µU/ml); G_0_ = fasting glucose levels (mmol/L); HOMA-IR = homeostasis model assessment of insulin resistance (µU**·**mmol)/(ml**·**L).

Rats were treated with three separate doses of lurasidone (0.2, 0.8 or 2.0 mg/kg), olanzapine (10 mg/kg) or vehicle. Values represented as means ± SEM at t = 30 min during the IGTT.

*indicates different from vehicle-treated animals, p<0.001.

**Figure 2:**
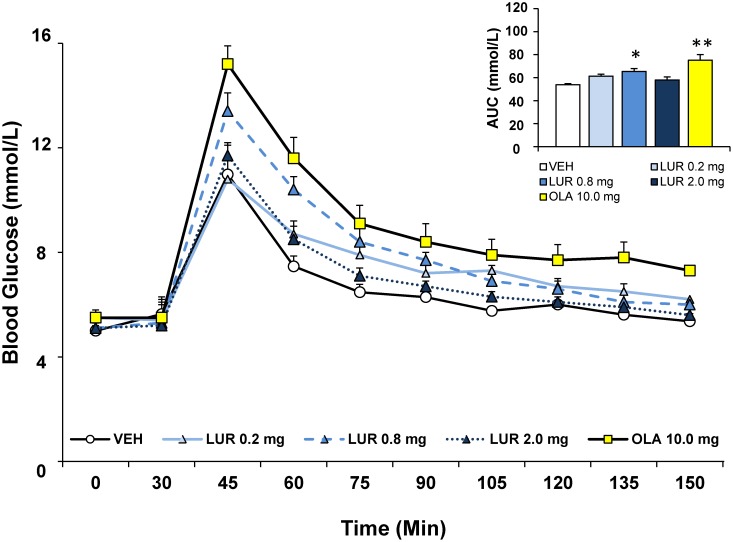
Acute effects of the SGA drugs lurasidone and olanzapine on glucose levels in adult female rats in the glucose tolerance test. Animals (n = 8 per group) received a single injection of vehicle, lurasidone (0.2, 0.8 or 2.0 mg/kg, s.c) or olanzapine (10 mg/kg, s.c.) in a volume 1 ml/kg. Glucose levels were recorded prior to drug treatment in overnight-fasted rats at Time 0, and then 30 minutes following drug administration (*x-axis*). Immediately following this glucose measurement, all rats were subjected to a glucose tolerance test by receiving an intraperitoneal challenge injection of 1 g/ml/kg of glucose, and blood glucose levels were measured every 15 minutes for the next two hours. Total cumulative glucose levels for each treatment group are summed as the “area under the curve” during the glucose tolerance test by graph inset (*top right*). Values represent group means ± SEM; *indicates different from vehicle-treated animals, p<0.01; **indicates different from vehicle-treated animals, p<0.001.

Following the glucose challenge, glucose levels rose rapidly in all animals, peaking in the first measurement after glucose injection (Figure 2). Peak glucose levels indicated a significant main effect of drug treatment [F_(4,40)_ = 5.60, p = 0.001], as glucose levels were significantly higher in the olanzapine (p<0.01) and lurasidone 0.8 mg/kg (p<0.05) group than in vehicle treated rats; there was no effect of the 0.2 mg/kg dose of lurasidone, and a slight (non-significant) increase in glucose levels caused by the 2.0 mg/kg dose of lurasidone. Similar results were obtained when the glucose area-under-the-curve was analyzed. A significant main effect of drug treatment [F_(4,40)_ = 9.07, p<0.001] and post-hoc analysis revealed a larger increase in glucose levels during the 120 minute test by olanzapine treated rats than vehicle (p<0.001; 39% higher levels than vehicle) and all other groups. The 0.8 mg/kg lurasidone treated group also showed significantly higher glucose levels compared to vehicle treated rats (p<0.01; 21% higher levels than vehicle) although unlike olanzapine, glucose levels were not significantly higher compared to the other two doses of lurasidone. The lower 0.2 mg/kg dose of lurasidone showed a non-significant trend towards higher glucose levels (p = 0.08; 14% higher levels than vehicle) compared to vehicle treated rats.

### HIEC

**Figure 3:**
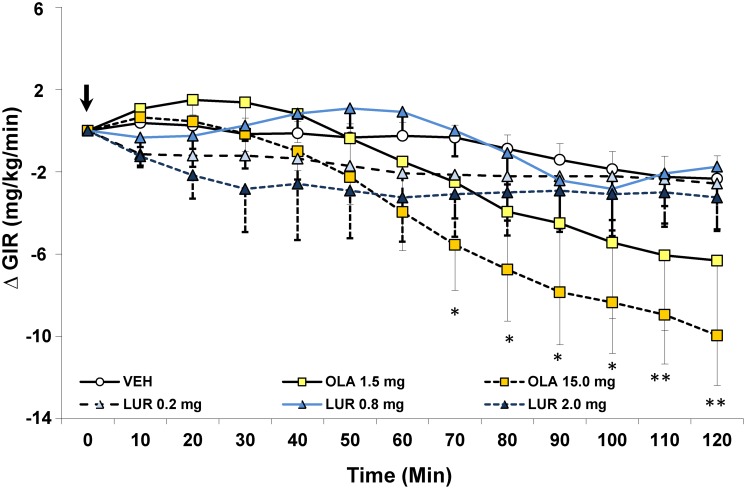
Acute effects of the SGA drugs lurasidone and olanzapine in adult female rats in the hyperinsulinemic-euglycemic clamp. Animals (n = 6–10 per group) were fasted overnight and subjected to the hyperinsulinemic-euglycemic clamp. After animals reached euglycemia (three consecutive blood glucose readings of 6.0±0.4 mmol/L), rats were treated with vehicle, lurasidone (0.2, 0.8 or 2.0 mg/kg, s.c) or olanzapine (1.5 or 15 mg/kg, s.c.) in a volume 1 ml/kg. Glucose levels were recorded every 10 minutes and the glucose infusion rate was adjusted as needed. Glucose infusion rates for each treatment group are presented as change in glucose infusion rate from euglycemia. Values represent group means ± SEM. *indicates different from vehicle-treated animals, p<0.05; **indicates different from vehicle-treated animals, p<0.01.

Average basal glucose levels were similar for all groups prior to euglycemia and administration of antipsychotic drugs (Figure 3). Insulin resistance during the HIEC is indicated by a reduction in the GIR, and therefore the primary analysis compared the effects of antipsychotics on change in the GIR over 120 mins. For the overall ANOVA, antipsychotic drug treatment was represented by between-subjects factors, while the change in GIR over time from the baseline value at t = 0 (i.e. at administration of the antipsychotic drug) was the within subjects factor. The ANOVA indicated a significant main effect of change in GIR over time [F_(12,516)_ = 18.70, p<0.0001], as well as an interaction of time×drug treatment [F_(60,516)_ = 2.92, p<0.0001]. Post-hoc analysis revealed that the interactive effect reflected a drug-selective effect of antipsychotic treatment on GIR over time, as insulin resistance increased selectively and gradually after the drug injection. By 70 minutes after antipsychotic drug injection, the olanzapine 15 mg/kg group displayed a significantly lower GIR than vehicle treated animals, which remained significant until the end of the test, and indicated the capacity of the drug to cause whole-body insulin resistance. The lower dose of olanzapine 1.5 mg/kg also caused a reduction in the GIR but this was never significant. By comparison, there was no effect at any time of the three different doses of lurasidone on the GIR.

## Discussion

The results of the current study provide the first published findings for the novel SGA drug lurasidone and its effects on the key metabolic indices of glucose tolerance and insulin resistance. Using the two separate techniques of the GTT and the HIEC in adult female rats, we were able to find converging evidence for a relatively benign metabolic profile for lurasidone. Across a tenfold range of doses, lurasidone caused modest but significant glucose intolerance with the single dose of 0.8 mg/kg. In the same GTT, there was no effect of any dose of lurasidone on insulin resistance measured by the HOMA-IR equation. These results were corroborated in the HIEC, when there was no effect of any of the three doses of lurasidone on the glucose infusion rate, indicating no evidence of insulin resistance. By contrast, olanzapine displayed a robust dose-dependent effect on glucose intolerance, as well as insulin resistance measured both HOMA-IR and in the HIEC.

The two procedures used in the current study provide complementary measures of metabolic physiology, and arguably represent the most reliable and accurate methods of determining glucose tolerance and insulin resistance. The GTT measures the ability of a fasted subject to return glucose levels to normal following a glucose challenge, and the procedure is commonly used in both preclinical and clinical studies of metabolic syndrome, prediabetes and Type 2 DM [43]. The GTT has been used in the diagnosis of Type 2 DM for decades, and provides a substantially more accurate determination of loss of glycemic control than measurement of fasting glucose levels only, which may misdiagnose Type 2 DM in at least one third of patients [44], [45]. The GTT is practical and shows strong cross-species homology, allowing precise evaluation of postprandial glucose regulation [46]. A limitation of the technique is its capacity to measure insulin resistance following a prandial/glucose challenge, as both glucose and insulin levels are free to vary. Insulin resistance is therefore best measured using “clamp” techniques, such as the HIEC, which provides an accurate index of cell-mediated glucose uptake via the action of insulin, and is therefore considered the “gold standard” test of insulin resistance [47]. We have previously noted a high (but not perfect) degree of correlation between results obtained with the GTT and the HIEC in rats treated with SGA drugs [48]. Our previous study indicated that the GTT may be slightly more sensitive at detecting metabolic dysregulation than the HIEC. This may explain why we observed glucose intolerance with a single dose of lurasidone, but no effect of the drug on insulin resistance measured by HOMA-IR or with the glucose infusion rate in the HIEC. The mechanisms underlying this possible differential sensitivity to the effects of antipsychotic drugs in the two procedures remains unknown. In the HIEC, insulin levels remain fixed at a high level for an extended period, whereas in the GTT levels of insulin are free to vary. Of relevance, a number of studies have determined that olanzapine can directly inhibit beta cell function, thereby attenuating insulin release in response to a glucose challenge [15], [49], [50]. It is feasible that olanzapine causes impairments in insulin release following the glucose challenge in the GTT that contribute to the elevated glucose levels in the test, and that these effects are more pronounced than the effects of insulin itself on glucose uptake in the HIEC; further study will be required to determine this possibility. The explanation of why glucose intolerance was observed with the intermediate, rather than highest, dose of lurasidone remains unknown. It is possible to speculate that the physiological mechanisms that underlie glucose intolerance may exert their effects in a non-linear manner, reflecting lurasidone’s complex pharmacological profile [51], but this will clearly require further study.

In the present study, we have chosen doses of drugs based on previous preclinical studies, while also considering known metabolic effects in humans when possible. The choice of dose requires some justification, as the metabolic effects of SGAs in humans can be dose-dependent [52]. The two doses of olanzapine represent a typical range used in metabolic studies in rats [25], [38]. The effects of the higher dose of olanzapine produced a 39% increase in the area-under-the-curve in the GTT. This is highly consistent with comparable human studies; for example, when Albaugh and colleagues treated non-patient subjects with a clinical dose of olanzapine, they observed a 42% increase in the area-under-the-curve of glucose levels in the GTT [18]. Interestingly, therefore, the dose of olanzapine in rats needed to reproduce equivalent metabolic effects in humans in the GTT is higher than would be expected, based on the known dopamine D2 receptor occupancy of the drug in the rat brain. A dose of 3 mg/kg of olanzapine is sufficient to occupy 65–70% of rat striatal D2 receptors [53], [54], which would represent the equivalent of receptor occupancy levels in patients at a therapeutic dose; however, the robust metabolic effects of the antipsychotic would not be evident at this dose in rats. While the interpretation of this finding requires further study, it may be possible that the metabolic effects of antipsychotic drugs are largely independent of central effects on D2 receptors, and that the physiological substrates that mediate glucose intolerance and insulin resistance are separate and proportionally less sensitive in the rat, requiring higher relative doses than central D2-mediated effects such as behavioral tasks [55] that model therapeutic human dosing. Comparing dosing, therefore, between two antipsychotic drugs where the metabolic effects of one in humans are unknown requires estimation and it is possible that the effects with lurasidone may be an underestimation.

As there are no previous reports of metabolic indices being measured with lurasidone, we chose doses of this drug based on behavioral and physiological studies and the effects of lurasidone in preclinical screens and models of schizophrenia. For example, Horiguchi and colleagues observed that while doses of 0.01 and 0.03 mg/kg (i.p.) of lurasidone were ineffective in reversing phencyclidine-induced cognitive impairment, doses of 0.1 and 0.5 mg/kg caused significant improvement in the task [56]. Doses of 0.25 and 0.5 mg/kg (i.p.), but not 0.1 mg/kg, of lurasidone increased dopamine efflux in the frontal cortex and hippocampus [57], which represents a physiological property common to SGA drugs. Our observation that there were no metabolic side-effects with the highest 2.0 mg/kg dose of lurasidone suggests that these effects were not present at a dose substantially higher than the average dose effective in behavioral studies.

The present observation that acute olanzapine treatment causes potent metabolic dysregulation is well established in the animal literature, and consistent with numerous previous rodent studies [25], [26], [38], [49], [58]–[65]. Also, as noted above, several recent studies in non-patient subjects have confirmed that acute olanzapine treatment in humans causes both glucose intolerance and insulin resistance in the absence of weight gain [18], [19]. This indicates that the SGA drug itself is directly causing metabolic dysregulation, rather than reflecting the influence of other potentially confounding variables that may be associated with schizophrenia, such as smoking [66], poor diet and lack of exercise [67]. We have previously reported that the magnitude of the metabolic effects of chronic treatment with olanzapine on a daily basis does not change over a period of months [37], [68], indicating that acute effects are an accurate predictor of chronic metabolic effects.

In contrast to the established effects of the SGA drug olanzapine on metabolic function, relatively little is known about the metabolic effects of lurasidone. To our knowledge, there are no preclinical studies of the metabolic effects of lurasidone. In the clinical literature, a number of studies have determined the effects of treatment with lurasidone on metabolic indices in clinical trials of the drug for treatment of schizophrenia and bipolar disorder. However, these measures have been limited to fasting glucose only, which - as noted above - provides a less accurate test of glucose or insulin dysregulation than the techniques used presently [44]. A recent systematic analysis of previous clinical trials with lurasidone reported that when effects were pooled from seven short term trials (≤12 weeks), there was a small but significant increase in fasting glucose levels versus placebo, which was described as “not…clinically meaningful” [69]. In clinical trials reported since this review, there is no evidence of lurasidone significantly increasing glucose levels versus placebo [70]. In clinical trials where patients are switched from other antipsychotic medications to lurasidone, small increases or decreases in fasting glucose levels have been reported, depending on the specific SGA at baseline [71]; for example, patients switched from aripiprazole to lurasidone showed an increase in fasting glucose levels of 5 mg/dL after 6 months of treatment, whereas those switched from olanzapine to lurasidone showed a decrease of 2 mg/dL. These minor changes in fasting glucose levels are consistent with the numerous reports of low metabolic liability for lurasidone with regards to its relatively low propensity for weight gain in patients [69]. Nevertheless, given the small but significant increase in fasting glucose reported in the analysis by De Hert and colleagues [69], as well as evidence from the current study that mild glucose tolerance can occur at certain doses following a glucose challenge, it may be worthwhile as a cautionary measure to follow up in lurasidone treated patients with additional study, using metabolic tests with greater reliability than fasting glucose levels, such as the GTT.

In summary, we have used separate tests of glucose tolerance and insulin resistance to determine the metabolic effects of the novel SGA lurasidone in adult female rats, using techniques that accurately model the metabolic side-effects of antipsychotic drugs in humans [20]. A limitation of the study is that our index of glucose infusion rates in the HIEC provided only a whole-body measure of insulin resistance. Pervious rat studies have used radioactive tracers to distinguish between the effects of antipsychotics on hepatic versus peripheral insulin sensitivity [49], [72], and while no effect of lurasidone was evident in the HIEC, it is possible that regional differences in insulin sensitivity may occur that were not detected with the current protocol. We observed dose-dependent, mild glucose intolerance, but no evidence of insulin resistance, indicating that the metabolic liability of lurasidone in humans is likely to be quite low, and certainly not of a comparable magnitude to a higher metabolic risk SGA drug such as olanzapine. However, given the large number of patients who may be potentially treated with lurasidone - particularly if the drug is tailored to the individual patient because of its weight sparing profile - and the harmful effects of uncontrolled glucose, a more thorough metabolic evaluation of the drug in humans would be justified.
